# A Diagnosis of Multiple Sclerosis Following Whiplash Injury: Is There a True Association?

**DOI:** 10.7759/cureus.13411

**Published:** 2021-02-18

**Authors:** Lauren Harris, Sofie Hateley, Aravindhan Baheerathan, Omar Malik

**Affiliations:** 1 Neuroscience, Imperial College Healthcare National Health Service Trust, London, GBR; 2 Neurology, Imperial College Healthcare National Health Service Trust, London, GBR

**Keywords:** multiple sclerosis, trauma, demyelination, whiplash

## Abstract

We report a case of a previously well, 25-year-old Caucasian female whose diagnosis of multiple sclerosis (MS) followed significant trauma. Her symptoms and signs developed quickly and satisfied the criteria for rapidly evolving relapsing-remitting MS. She was started on natalizumab (Tysabri) and was stabilized. We discuss the existing literature on traumatic demyelination and possible underlying mechanisms.

## Introduction

Since the first descriptions of multiple sclerosis (MS), it has been suggested that trauma may have a role in causation or exacerbations [1]. The link between MS and trauma is yet to be fully established. However, the association between trauma and demyelination has important implications for the patient, in terms of disability, the physician, and medicolegally. Case-controlled studies support a role for trauma in triggering demyelination but cohort studies support the counter view [1-3]. The most frequent form of trauma responsible for the appearance or recurrence of MS is a whiplash injury, often from a rear-end collision [4].

We report the case of a previously fit and well 25-year-old female who developed rapidly evolving relapsing-remitting MS following a rear-end vehicle collision only a few days earlier. Her demyelinating disease involved the spinal cord and brainstem. We discuss the possible biological mechanisms that may underlie demyelination precipitated by physical trauma.

## Case presentation

A previously fit and well, 25-year-old female was involved in a vehicle collision, where she was shunted at 20 miles per hour. This collision caused her car to spin 180 degrees and her body to jolt forward. There was no loss of consciousness or immediate injuries. In the following hours, she developed upper and lower back pain, typical of a soft tissue injury (whiplash). Ten days later she reported ‘dizziness’ and had paroxysmal positional vertigo which progressed to chronic vertigo, intermittent diplopia, vomiting, and dehydration, necessitating hospital attendance and admission. Five weeks post-injury, she developed typical Lhermitte’s and exertion related lower limb weakness (Uhthoff’s phenomenon where symptoms worsened with increased body temperature on exercise). At ten weeks, she developed pins and needles in her left hand but by week eleven she was walking normally again, without treatment, and had a neurological review.

She reported no past medical history, corroborated by both GP and hospital records, and no recent infections. Medication history included the oral contraceptive pill and occasional vitamin supplements. She was a non-smoker, non-drinker, with no history of recreational drugs, or significant travel. Of note, she had an extensive family history of MS with maternal and paternal inheritance, having at least two first degree relatives with a diagnosis of MS.

On examination (11 weeks post-injury), she had bilateral internuclear ophthalmoplegia, globally brisk deep tendon reflexes, bilateral extensor plantar responses, and dorsal column sensory disturbance in her lower limbs to her anterior superior iliac spines. The initial differential diagnosis was that of inflammatory demyelination, though a possible traumatic disc prolapse causing cervical cord compression could have accounted for the myelopathic signs.

Investigations

**Figure 1:**
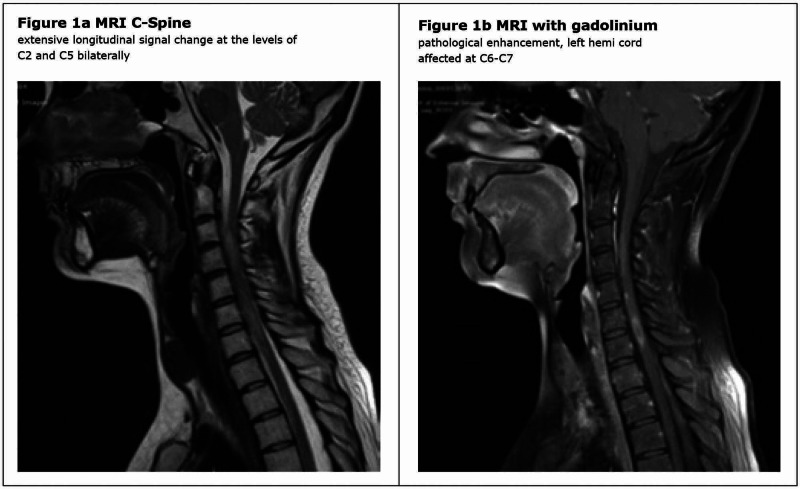
MRI cervical spine - four weeks post injury

**Figure 2:**
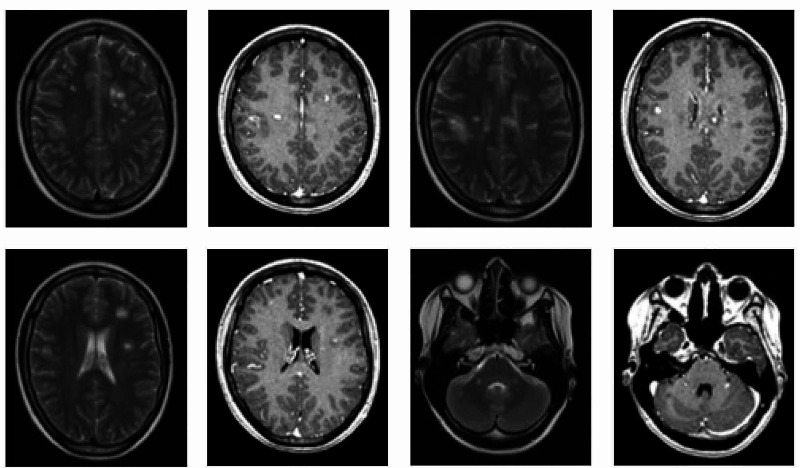
MRI brain - four weeks post injury; T2 weighted and T1 weighted with contrast at different axial cuts

**Figure 3:**
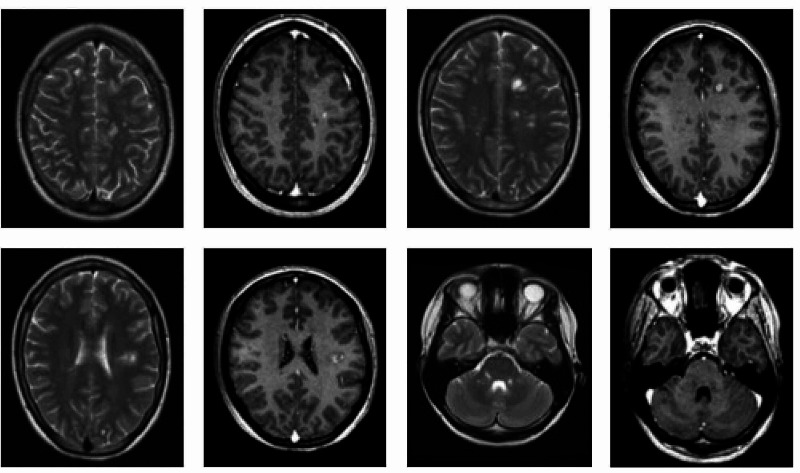
MRI brain - 12 weeks post injury; T2 weighted and T1 weighted with contrast at different axial cuts

First neuro-axial imaging was done at four weeks post-injury and interval imaging at week 12. This revealed widespread neuro-inflammatory disease throughout the central nervous system. The first MRI of the spine, four weeks post-injury, showed evidence of extensive longitudinal signal change and multiple enhancing lesions in the cervical cord (Figure 1). The MRI of the brain at that time identified multiple foci of signal hyperintensity throughout the parenchyma, both in the infratentorial and supratentorial compartment (Figure 2). Some lesion enhancement was seen, consistent with an active disease process, as well as some non-enhancing lesions, suggesting dissemination in time (Figure 2). The repeat imaging at week 12 confirmed the presence of dissemination of time and space, with the identification of new enhancing lesions, meeting the McDonald criteria for a diagnosis of MS (Figure 3). Visual evoked potentials, with responses to monocular ‘full field’ stimulation from three occipital electrodes, were normal (latency, amplitude, and waveforms). Cerebrospinal fluid (CSF) examination revealed normal protein and cell count, with selective intrathecal oligoclonal bands.

Management

Her initial treatment was 2 g IV methylprednisolone followed by a steroid taper. In view of her highly active presentation clinically and radiologically, she was commenced on a monthly natalizumab (Tysabri) 300 mg intravenous infusion, and has remained clinically and radiologically stable for three years (JC virus negative).

## Discussion

The literature on the association between physical trauma and onset of MS, consisting of interview-based case-control studies, case reports, and cohort studies, is contradictory. This is in part related to small sample sizes, using a broad definition of trauma (e.g. fractures, burns, head injury, spinal injury), and grouping different ages together (e.g. children with adults) [1]. Meta-analyses of case-control studies have shown a significant association between head trauma and the risk of MS [1,3]. The most robust analysis of 36 case-controlled studies (5922 MS cases) showed a statistically significant relationship between head trauma and MS diagnosis, including on exclusion of low-quality studies [1]. Cohort studies have not demonstrated this association, although numbers are small and consist of low-quality studies [1-3]. A population-based, longitudinal follow-up study in 2015 showed that patients with spinal cord injury have an increased risk of developing MS [4]. The most frequent form of trauma putatively associated with the appearance or recurrence of MS is the whiplash injury, often from a rear-end collision [5].

There are a number of criticisms of the studies to date. First, there are a limited number of studies, particularly recent ones, and, due to the nature of both MS and trauma, there are no prospective randomized methods in a controlled setting. Second, the majority of studies are uncontrolled case series or reports with small sample sizes [1]. Third, even well-designed retrospective case-controlled studies suffer recall bias. Fourth, the definition of trauma is not standardized and the specifics of trauma are not detailed [1]. MS is an unpredictable and variable disease, with different genetic, immunological, clinical, and pathological involvement, making such investigations difficult.

The breakdown of the blood-brain barrier is an early event in the pathogenesis of an MS lesion, demonstrated by serial MRI and positron emission tomography studies [6]. Clinical, neuropathological, neuropsychological, radiological, and experimental evidence has shown that this breakdown can occur following physical trauma to the head, neck, or upper back, including mild concussive and whiplash injuries [7]. It has been suggested that mild trauma, and the resulting increased permeability of the blood-brain barrier, can lead to the formation of new lesions or the enlargement and activation of old ones. Concussion can cause diffuse microscopic lesions of blood vessel walls [6]. The postulated mechanism of breakdown of blood-brain barrier allowing trafficking of T-lymphocytes into previously immune-privileged areas of the central nervous system has been demonstrated experimentally [8]. Physical trauma may activate an otherwise dormant MS, by activating an underlying, and possibly inherited, defect in the small blood vessels of the brain [1]. Furthermore, the role of trauma exacerbating focal myelin damage has been demonstrated histologically in other non-immune myelin disorders [9]. In this case, there is a strong family history of MS, and we speculate that this proposed mechanism may apply, although with regret T-lymphocyte concentrations were not formally assessed (the CSF had a normal cell count).

Physical trauma can lead to focal areas of myelin damage as well as the breakdown of the blood-brain barrier. The latter could allow the exposure of previously hidden myelin antigens to be seen by immune cells and thus trigger a “secondary” autoimmune phenomenon. This process has been demonstrated in the nervous system with “sympathetic ophthalmia” due to retinal damage and exposure to retinal antigens [10].

Within the literature, the time course between trauma and onset of symptoms has not been fully characterized. In this case, a previously well patient developed neurological symptoms ten days following trauma and deteriorated rapidly. She had no pre-existing symptoms of MS. Studies report cases of MS beginning within two or three months of trauma, others, including the earliest association by Mendel in 1897, reported cases within a year [2]. Whilst the time course is disputed, most authors agree that symptoms develop within a year of trauma [11]. As the onset of MS is determined with an accuracy of one calendar year, some argue that this association can be attributed to increased attention to symptoms after an injury [2]. In our case, with such dramatic symptoms developing so fast, we argue that this is not so.

This study has a number of limitations. It is a single case report. The patient had had no MRI scans prior to the trauma. It could be suggested that the patient may have already had radiographical lesions. However, MS is a clinical diagnosis, and she was completely symptom-free prior to the whiplash injury. We believe that in this case, the trauma was the trigger that activated MS, adding to the body of literature supporting this view.

## Conclusions

This case suggests an association between trauma and the activation of MS in a predisposed individual. More rigorous studies are needed to establish whether there is an association of trauma with MS, whether trauma triggers MS, and to explore the time course between trauma and symptom onset. Prospective studies, with large sample sizes, and long follow-up times are necessary. It is important to have a standardized definition of physical trauma, provide specific details, and have a validated measure of severity to assess causal links between trauma and MS.
